# Transmission risk of Oropouche fever across the Americas

**DOI:** 10.1186/s40249-023-01091-2

**Published:** 2023-05-06

**Authors:** Daniel Romero-Alvarez, Luis E. Escobar, Albert J. Auguste, Sara Y. Del Valle, Carrie A. Manore

**Affiliations:** 1 Biodiversity Institute and Department of Ecology & Evolutionary Biology, University of Kansas, Lawrence, KS 66044 USA; 2 Information Systems and Modeling (A-1), Los Alamos National Laboratory, Los Alamos, NM USA; 3 OneHealth Research Group, Facultad de Medicina, Universidad de las Américas, Quito, Ecuador; 4 Department of Fish and Wildlife Conservation, Virginia Tech, Blacksburg, VA 24061 USA; 5 Center for Emerging, Zoonotic, and Arthropod-Borne Pathogens, Virginia Tech, Blacksburg, VA 24061 USA; 6 Department of Entomology, Fralin Life Science Institute, College of Agriculture and Life Sciences, Virginia Tech, Blacksburg, VA 24061 USA; 7 Theoretical Biology and Biophysics (T-6), Los Alamos National Laboratory, Los Alamos, NM USA

**Keywords:** Oropouche virus, Oropouche fever, Spatial modeling, Hypervolumes, Distribution modeling, Risk mapping, One-class support vector machines, Convex-hulls

## Abstract

**Background:**

Vector-borne diseases (VBDs) are important contributors to the global burden of infectious diseases due to their epidemic potential, which can result in significant population and economic impacts. Oropouche fever, caused by Oropouche virus (OROV), is an understudied zoonotic VBD febrile illness reported in Central and South America. The epidemic potential and areas of likely OROV spread remain unexplored, limiting capacities to improve epidemiological surveillance.

**Methods:**

To better understand the capacity for spread of OROV, we developed spatial epidemiology models using human outbreaks as OROV transmission-locality data, coupled with high-resolution satellite-derived vegetation phenology. Data were integrated using hypervolume modeling to infer likely areas of OROV transmission and emergence across the Americas.

**Results:**

Models based on one-support vector machine hypervolumes consistently predicted risk areas for OROV transmission across the tropics of Latin America despite the inclusion of different parameters such as different study areas and environmental predictors. Models estimate that up to 5 million people are at risk of exposure to OROV. Nevertheless, the limited epidemiological data available generates uncertainty in projections. For example, some outbreaks have occurred under climatic conditions outside those where most transmission events occur. The distribution models also revealed that landscape variation, expressed as vegetation loss, is linked to OROV outbreaks.

**Conclusions:**

Hotspots of OROV transmission risk were detected along the tropics of South America. Vegetation loss might be a driver of Oropouche fever emergence. Modeling based on hypervolumes in spatial epidemiology might be considered an exploratory tool for analyzing data-limited emerging infectious diseases for which little understanding exists on their sylvatic cycles. OROV transmission risk maps can be used to improve surveillance, investigate OROV ecology and epidemiology, and inform early detection.

**Supplementary Information:**

The online version contains supplementary material available at 10.1186/s40249-023-01091-2.

## Background

Vector-borne diseases (VBDs) account for at least 17% of the total infectious disease burden worldwide and the yearly loss of approximately 52,000 disability adjusted life years [1]. VBDs concentrate in the tropics but climate change, globalization, and landscape conversion have facilitated their expansion [2]. Novel and invasive emerging pathogens challenge global health security and public health intelligence due to the limited understanding of the ecological and epidemiological drivers of their transmission.

The Neotropics are understudied in terms of endemic and emerging diseases [1]. Many endemic VBDs in Latin America are considered neglected with nil to poor epidemiological surveillance [3]. A notable emerging zoonotic disease that remains poorly understood is Oropouche fever, caused by Oropouche virus (OROV), first described in Trinidad and Tobago in 1954. Oropouche fever presents as a syndrome clinically indistinguishable from other VBDs such as dengue, Zika, or Mayaro fevers, with symptoms commonly including fever, headache, and myalgia [4, 5].

OROV has infected more than 500,000 people across Latin America, especially in Brazil and Peru, and these numbers are known to be gross underestimations [6]. OROV is a tri-segmented negative-sense RNA virus, taxonomically classified into the genus *Orthobunyavirus*, family Peribunyaviridae. OROV is maintained in its sylvatic cycle by wildlife hosts and arthropod vectors. Although knowledge is limited, reports suggest that non-human primates (e.g*., Callithrix penicillata*) and sloths (e.g., *Bradypus tridactylus*) play a role as hosts [4]. Once OROV spills over from wildlife into human populations, it is transmitted mainly via the midge *Culicoides parensis* and potentially also by *Culex quinquefasciatus*, a mid-size mosquito found globally [4, 7–9].

Given the epidemic potential of OROV, the limited data on the disease system, and the recent and more frequent outbreaks outside endemic areas [10], there is a need to identify regions for likely OROV spread to human populations [1]. We estimated the geographic potential of OROV in the Americas through a biogeographic risk mapping framework using hypervolume models, satellite-derived landscape data, and OROV human case data [11, 12]. A series of modeling protocols were assessed to identify the modeling approaches with robust descriptive and predictive capabilities. We used these models to identify areas where OROV may emerge and where cases of unknown febrile syndromes could be attributed to OROV. We also studied the role of landscape degradation on OROV emergence and estimated the amount of people at risk.

## Methods

### Study design

**Fig. 1:**
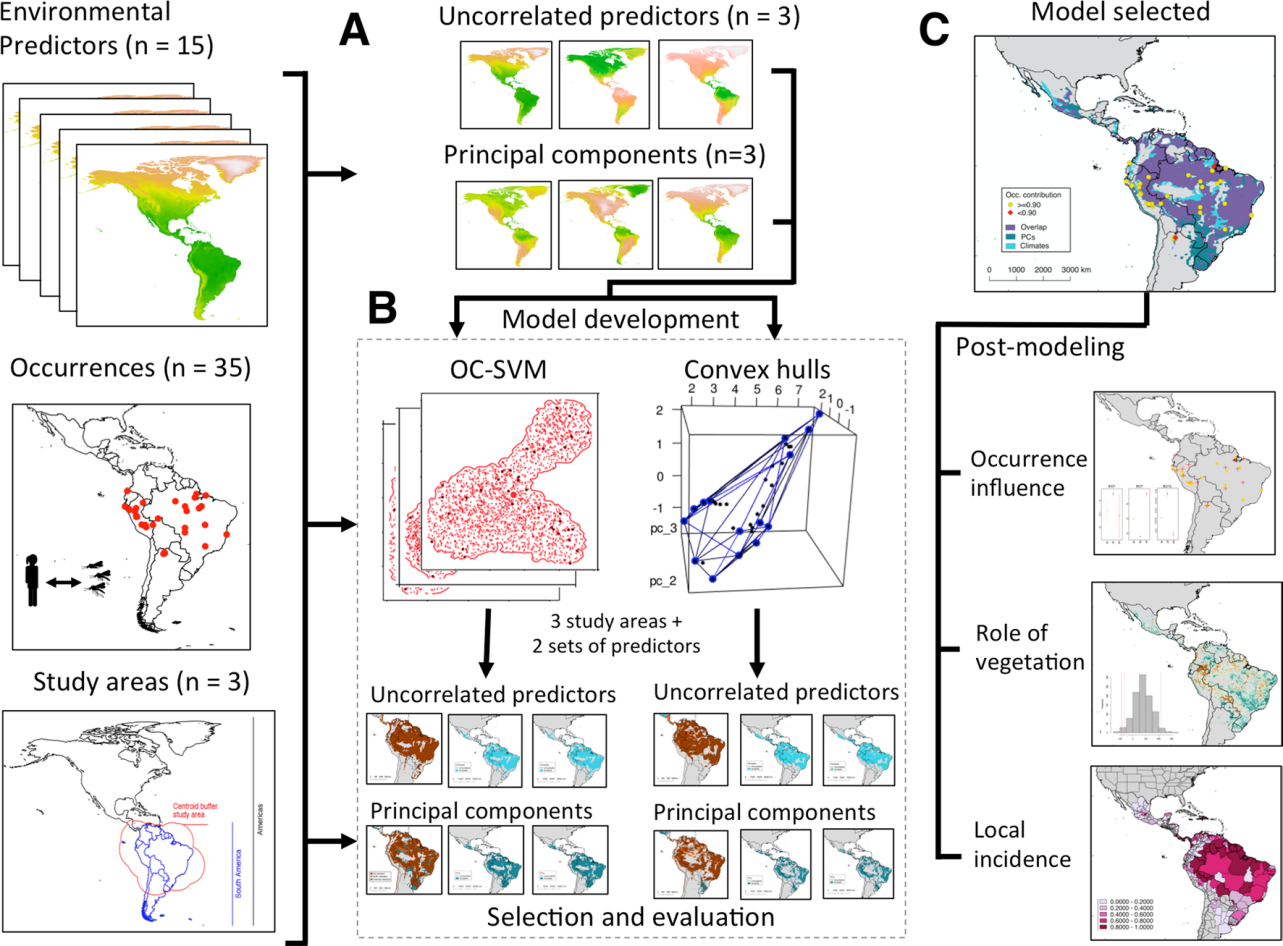
Summary of the modeling and post-modeling steps followed for this research. We coupled 35 curated occurrence records of human Oropouche fever outbreaks with 15 environmental predictors for model development (**A**). Environmental multicollinearity was treated via a correlation matrix to select three environmental predictors (i.e., BIO1, BIO7 and BIO12), and an independent principal component analysis (PCA) over the 15 original variables for a total of two sets of predictors for model development over three different model calibration regions (**A**). We used one-class support vector machines (OC-SVM) and convex hull hypervolumes as algorithms to explore the environmental and geographical space defined by the occurrences and environments processed (**B**). After model selection and evaluation, we examined (i) the influence of each occurrence in the geographic space, (ii) the role of vegetation difference on recorded outbreaks, and (iii) calculated the human population overlapping with the Oropouche virus (OROV) transmission risk map (**C**)

We followed an analytical framework based on niche theory [11–13]. We mapped the potential distribution of OROV by employing species distribution hypervolume models to predict where OROV infections are more likely to occur based on environmental features. Models were based on environmental interpolations and calibrated with information from localities where human infections have been notified. [14–16]. Our entire approach is summarized in Fig. 1, includes collection, curation, and standardization of Oropouche reports, manually inspected to include only those representing confirmed OROV diagnosis starting 2000s to match contemporary environmental predictors (see “Occurrences” section). We controlled for multicollinearity from environmental predictors using two methods of variable reduction: correlation matrices and principal component analysis (PCA; see “Environmental predictors” section). Due to the sensitivity of species distribution models to the calibration region, we assessed three different calibration regions to capture uncertainty (see “Model calibration region” section). Two algorithms were examined—one-class support vector machines (OC-SVM) and convex hull hypervolumes (See “Model calibration, evaluation and selection” section). Finally, the best model was further processed to determine the role of vegetation on disease emergence, and the amount of people living within risk areas (see “Post-modeling” section; Fig. 1).

### Occurrences

**Fig. 2:**
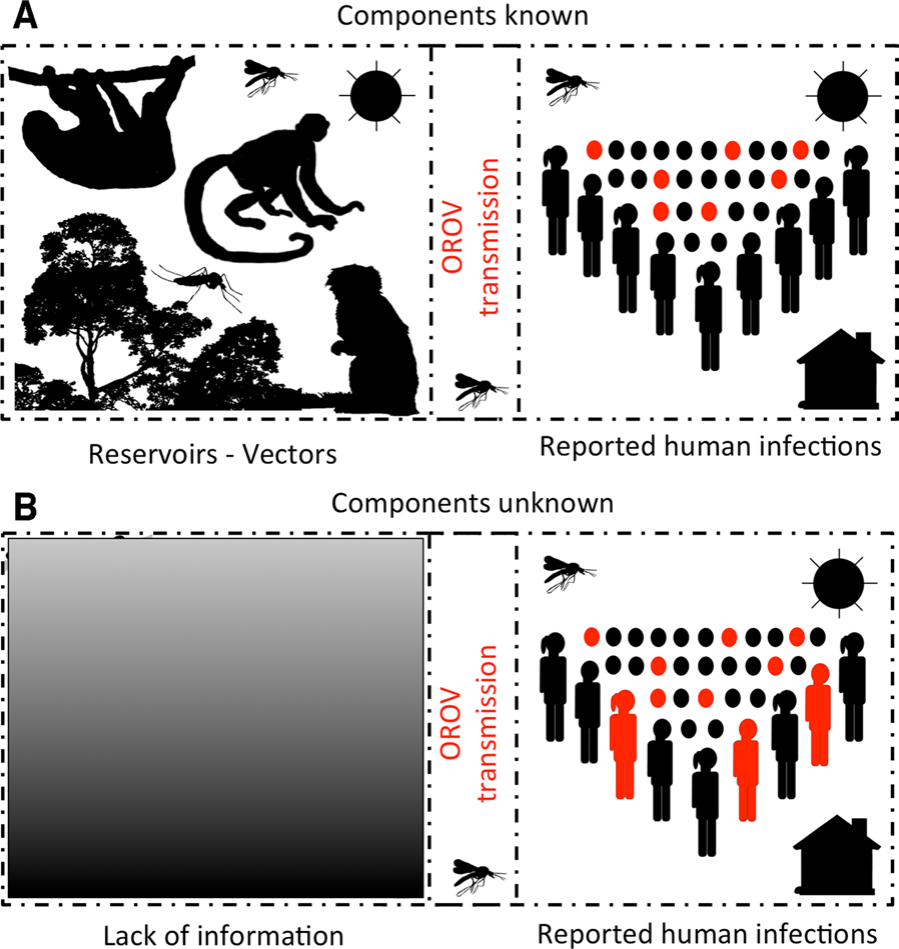
Schematic representation of component or black box-based strategies for infectious disease species distribution modeling. In well-known systems, disease models should aim to model each component driving the life cycle of the pathogen to better characterize its distribution (**A**; [11, 12]). However, for Oropouche virus (OROV), there are multiple gaps in knowledge to actually make assumptions about its sylvatic cycle, specifically, reservoirs and vectors driving epizootics are poorly represented in the scientific literature (**B**; [4]). For these cases, the presence of human outbreaks allows a black box modeling where we assume that detected human cases represent the manifestation of the entire virus cycle despite the unknowns surrounding its components. Silhouettes developed with Adobe Photoshop Elements

Records for confirmed human outbreaks were compiled following Romero-Alvarez & Escobar [4] and complemented by recent reports [17–21]. We analyzed records for human cases due to the uncertainty of vectors involved in the sylvatic transmission of OROV and the lack of information on wildlife hosts of the virus [4, 22]. Thus, we assumed that the presence of the disease in human populations represent the presence of all components of the disease system that allow successful spillover transmission from wildlife to humans in a particular region. This modeling strategy followed the ‘black box’ approach used in disease-risk mapping (Fig. 2; [11, 12, 23]).

We constrained the analysis to human cases (henceforth OROV occurrences) starting from the 2000s to match the timeframe of our selected environmental predictors (see below). We trimmed the database to eliminate duplicate records and avoided spatial autocorrelation by imposing a distance filter of 20 km as proxy of likely vector dispersal via the *SpThin* R package [24, 25]. We ended with 35 OROV occurrence records across South America for further analysis (Additional file 1).

### Environmental predictors

We coupled OROV records with climatic predictors at ~ 7 km resolution from MERRAclim, a satellite-derived data repository of temperature and humidity [26]. We used 15-bioclimatic MERRAclim predictors avoiding those combining temperature and humidity at the same time to avert potential artifacts (i.e., BIO8-9, BIO18-19; [27]). From this initial set of climatic variables we obtained two sets of predictors. First, we built a Pearson correlation matrix and selected three uncorrelated predictors for model development: annual mean temperature (BIO1), temperature annual range (BIO7), and annual mean specific humidity (BIO12; Fig. 1 and Additional file 2); these predictors are known to set important constrains on the distribution of multiple species including *Culicoides paraensis* and different dipteran vectors [28–31]. Second, we applied a principal components analysis (PCA) on the 15 MERRAclim variables and used the resulting principal components (PCs) recovering more than 90% of information [11], to characterize the contribution across the 15 environmental predictors in few variables avoiding multicollinearity [32]. PCAs were developed across diverse study areas using the *kuenm* package [33].

### Model calibration region

One of the key parameters driving model outputs in species distribution and ecological niche models is the definition of the dispersal capacity of the species, or ***M*** parameter (sensu [34, 35]). ***M*** consistently affects model outputs in terms of predictive performance metrics [36, 37]. Although the importance of ***M*** has been highlighted thoroughly, we lack a standard methodology to define it, and current approaches overlook biological realism ([34–39]; but see [40]). To capture uncertainty in our ***M*** definition as study area for model calibration, we used three model calibration regions: (i) a buffer developed with the mean of distances from each occurrence to the centroid [41], (ii) continental South America, and (iii) the entire Americas continent (Fig. 1 and Additional file 2).

### Model calibration, evaluation, and selection

#### Modeling methods

We generated models using two hypervolume presence-only algorithms that take advantage of the environmental similarities between OROV occurrences and other regions of a user-defined environmental space [11, 12, 42]. First, we developed hypervolumes via one-class support vector machines (OC-SVM; [42]), which builds hyperellipses around the observed occurrences in environmental space using a uniform distribution. Then, OC-SVM trims the environmental space and leaves the regions that enclose all the available occurrences. We used the algorithm parameters following the software recommendations (i.e., *µ* = 0.01 and ***γ*** = 0.5) to obtain a tighter environmental distribution across OROV occurrences [42]. Models were calibrated and transferred to geography via the ‘*hypervolume_svm*’ and ‘*hypervolume_project*’ functions available in the *hypervolume* package in R [43]. Second, we constructed convex hulls in the environmental space using the *‘convhulln’* function in the *geometry* package in R [44]. Via convex hulls, OROV occurrences delimit a multidimensional polygon focusing on the marginal occurrences in the environmental space [11]. We developed OC-SVM and convex-hull models using the PCs and the three original environmental predictors on each model calibration region (Fig. 1). Because each OROV occurrence in the environmental space could represent many sites in geography (i.e., Hutchinson duality [16, 45]), models were projected to their respective geographies for spatial interpretation. Considering the multiple unknowns regarding OROV sylvatic cycle and the limited amount of data regarding human outbreaks for the modeling, we decided to avoid using data-hungry algorithms such as Maxent and focus on the interpolative capabilities of the hypervolumes selected to prevent uninformative extrapolations [46].

#### Model evaluation

Model evaluation for presence-only data is challenging [36, 47, 48]. Ideally, it should be done using independent datasets that allow the discrimination of omission and commission errors [11, 49]. However, for infectious diseases, independent datasets are seldom available, are tainted with misdiagnosis (i.e., other disease), lack of confirmation (i.e., immunological tests), or lack pathogen identification (i.e., only clinical diagnosis; [50]). For the particular case of OROV, a recent study found that the gold-standard primers for identification of the pathogen were unable to correctly detect OROV cases [51]. As such, our evaluation method used the whole available OROV dataset by splitting data in calibration and evaluation sets.

We used a bootstrap approach to assess the ability of randomly selecting 70% of OROV occurrences (calibration dataset) to predict the other 30% (evaluation dataset; [52, 53]). We implemented this approach in the environmental and geographic space defined by the two hypervolumes. We averaged the score from 50 replicates (i.e., the mean performance of occurrence predicted = 1, unpredicted = 0) to obtain an ensemble model as a metric of model agreement [52, 54–56]. We accounted for variation examining the 2.5 and 97.5 percentiles of the distribution of the model ensemble (Additional file 2). Models were then projected to geography in the form of OROV-transmission risk maps ([11]; Fig. 1). Because our evaluation was intended to be independent of the dispersal potential of OROV (i.e., ***M***; [35, 46]), we avoided metrics based on the presence of unsuitable pixels like Pearson et al. *P*-value or the binomial probability test [11, 16, 57].

### Post-modeling

#### Influence of occurrences on geography

We tested the extent to which each OROV occurrence affected the final transmission risk map using a Jackknife approach. We built models with *n*-1 points and measured the proportion of risk-area estimated. For this analysis, occurrences decreasing the total amount of pixels more than 10% were identified as those that ‘if-left-out’ would be more impactful for the overall geographical output. We extracted the mean values for the environmental variables using a 100 km buffer around the more impactful occurrences and compared these values against the mean of the values obtained across all OROV occurrences.

#### Vegetation cover and OROV outbreaks

We explored the extent to which vegetation cover in OROV occurrence localities differed from random locations. For this analysis, we used the normalized difference vegetation index (NDVI) and the enhanced vegetation index (EVI). Vegetation indices were obtained from the MOD13A2 (version six), 16 days rasterized products at 1 km from the Moderate Resolution Imaging Spectroradiometer (MODIS) instrument on board of the TERRA satellite [58]. We calculated the average of NDVI and EVI values of 2003 and 2019 and obtained the difference between these images using Google Earth Engine [59] to depict vegetation cover change in time. Then, we developed a randomization test based on the mean and the median of NDVI and EVI values of the 35 OROV occurrences against a null distribution built with 1000 replicates of 35 random draws across the geographic area predicted as of risk for OROV as described above [60, 61].

#### Human populations at risk

We estimated the human population at risk as the sum of all population per pixel overlapping with the OROV risk maps using population gridded estimates for 2020 [62]. We repeated this analysis for each province, department, or state in the Americas and obtained a proxy of incidence by dividing the population number on suitable pixels per province, department, or state by the total number of population pixels in each province, department, or state. We depicted this local incidence via a choropleth map showing low or high population at risk of OROV (Fig. 1). All analyses were performed in R software (R core team; version 3.6.3–2020; Vienna, Austria) using the available functions and packages listed in the additional file 3 [63]. Scripts to replicate this experiment can be found in https://github.com/daromero-88/OROV-transmission-risk-models-.

## Results

### Model performance

In the environmental space, hypervolumes based on OC-SVM outperformed the predictability of OROV occurrences compared to convex hulls irrespective of the set of predictors (original vs. PCs) or the model calibration region employed (Table 1). In the geographic space, OC-SVM hypervolumes developed with PCs showed a better performance only while using the Americas as calibration region, conversely, when using climates as predictors, OC-SVM hypervolumes performed better across the three calibration regions (Table 2). Performance in the geographic and environmental space was consistent when using convex hulls, independent of the predictors used to build the models (Tables 1 and 2).

**Table 1:** Performance metrics of hypervolumes in the environmental space

Environmental predictors	Model calibration region	Mean performance in E (*SD*)		Total volume	
		OC-SVM	Convex hulls	OC-SVM	Convex hulls
PCs prop. = 95%	Centroid based buffer	0.70 (0.12)	0.54 (0.17)	80.25	87.16
PCs prop. = 95%	South America	0.68 (0.11)	0.56 (0.18)	50.55	61.04
**PCs** **prop. = 96%**	**Americas**	**0.76 (0.15)**	0.45 (0.19)	20.04	15.32
Climates	Centroid based buffer	0.74 (0.11)	0.44 (0.17)	8,426,855	10,131,022
Climates	South America	0.74 (0.13)	0.48 (0.20)	8,444,272	10,131,022
Climates	Americas	0.72 (0.13)	0.51 (0.18)	8,507,409	10,131,022

Performance metrics for one-class support vector machines (OC-SVM) and convex hulls hypervolumes were measured in the environmental (E) space using three different model calibration regions and two categories of environmental predictors. Best performing model in bold. *SD* Standard deviation, *PCs* Principal components, *Prop.* Cumulative proportion of the three principal components used for model calibration

**Table 2:** Performance metrics of hypervolumes in the geographic space

Environmental predictors	Model calibration region	Mean performance in G (*SD*)		Total suitable pixels	
		OC-SVM	Convex hulls	OC-SVM	Convex hulls
PCs prop. = 95%	Centroid based buffer	0.49 (0.17)	0.54 (0.17)	96,335.5	83,375.5
PCs prop. = 95%	South America	0.48 (0.16)	0.56 (0.18)	91,830	80,161
**PCs prop. = 96%**	**Americas**	**0.63 (0.17)**	0.45 (0.19)	122,456	79,344
Climates	Centroid based buffer	0.62 (0.16)	0.44 (0.17)	114,231	107,553.5
Climates	South America	0.60 (0.17)	0.48 (0.20)	114,917	105,012
Climates	Americas	0.58 (0.20)	0.51 (0.18)	122,851	120,561

Performance metrics of one-class support vector machines (OC-SVM) and convex hulls hypervolumes were measured in the geographical (G) space using three different model calibration regions and two categories of environmental predictors. Best performing model in bold. *SD* Standard deviation, *PCs* Principal components, *Prop.* Cumulative proportion of the three principal components used for model calibration

### Calibration region and model outputs

Both the calibration regions and the predictors employed influenced the volume of environmental conditions predicted in relation to the OROV occurrence records. For instance, OC-SVM hypervolumes and convex hulls coupled with PCs showed an inverse relationship between calibration region and volume: the larger the calibration region, the smaller the environmental space predicted by the model (Table 1). On the contrary, while creating OC-SVMs with climatic predictors, the volume increased proportionally with the model calibration region. For convex hulls and climatic predictors, the volume remained stable (Table 1). These relationships were subtly reflected in the geographic space when using either hypervolume and mostly unnoticeable when using climatic predictors (Additional file 2).

### Model selection

**Fig. 3:**
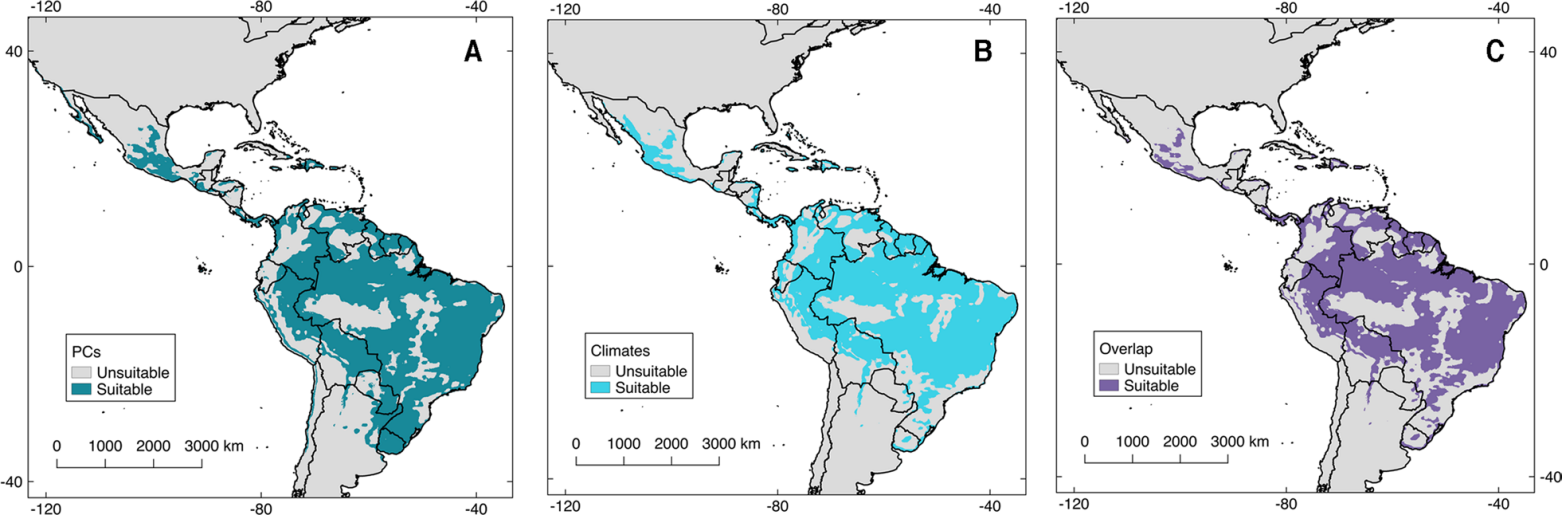
Potential distribution of Oropouche virus (OROV) based on one-class support vector machines (OC-SVM) hypervolumes. Models based on one-class support vector machines hypervolumes and calibrated in the Americas had the best performance metrics, the larger geographical prediction, and the best agreement between suitability of principal components (PCs; **A**) and climatic predictors (**B**). The map in panel **C** shows areas of overlap between the suitability of both environmental predictors. Shapefile of the Americas obtained from NaturalEarth (https://www.naturalearthdata.com/) and maps developed with QGIS 2.18 ‘Las Palmas’ and Adobe Photoshop Elements

**Fig. 4:**
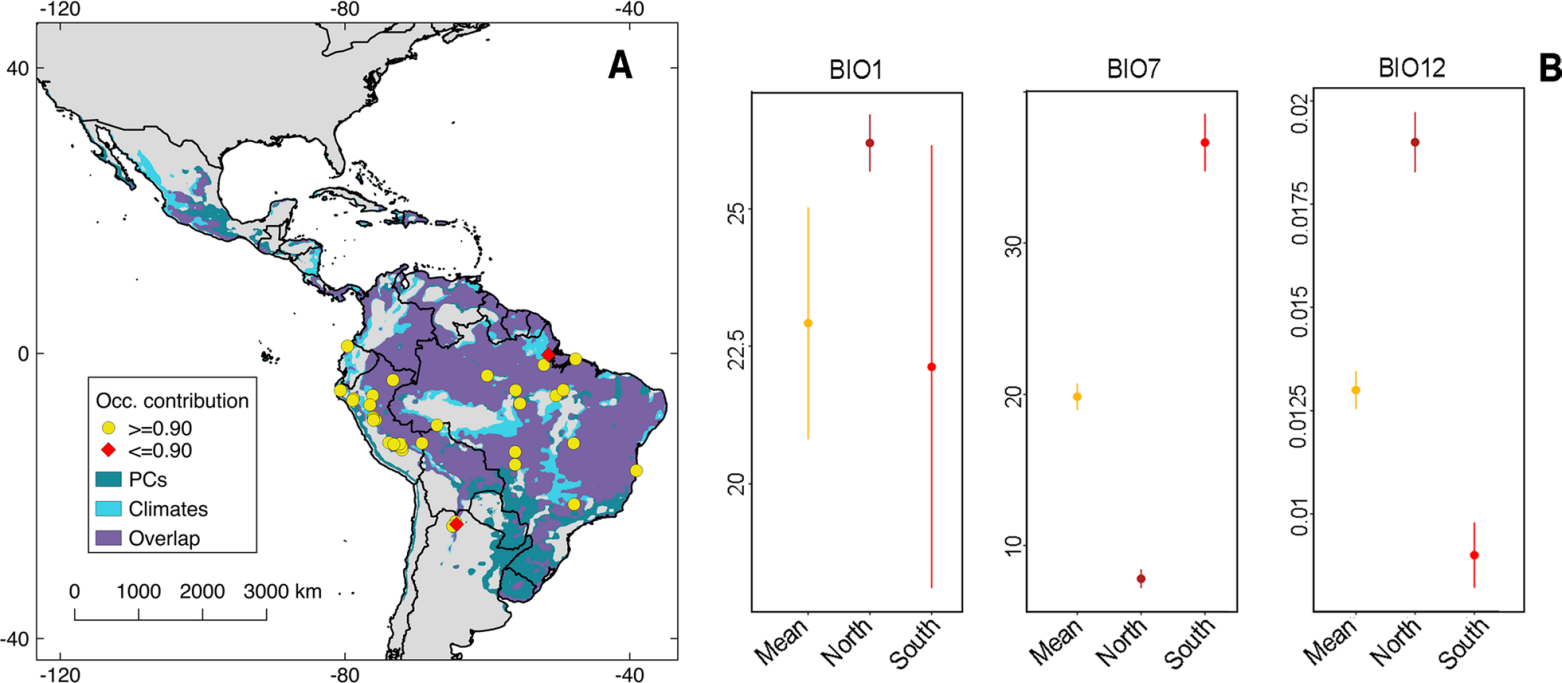
Occurrence contribution to the Oropouche virus (OROV) transmission risk map. Two occurrences (red; **A**) decreased the percentage of prediction in more than 10%. The localities identified differed climatically from the average of the rest of the points especially for BIO7 and BIO12 (**B**). BIO1: Annual mean temperature; BIO7: Temperature annual range; BIO12: Annual mean specific humidity. Shapefile of the Americas obtained from NaturalEarth (https://www.naturalearthdata.com/) and maps developed with QGIS 2.18 ‘Las Palmas’ and Adobe Photoshop Elements

Models developed with OC-SVM hypervolumes for the Americas were considered the best following multiple criteria: (i) good descriptive performance in the environmental and geographic space (Tables 1 and 2), (ii) agreement between model calibration regions and environmental predictors (Additional file 2), (iii) increased geographic prediction when combining climatic predictors and PCs (i.e., available suitable pixels: hypervolumes = 67.41% vs convex hulls = 48.23%), (iv) and low uncertainty (Additional file 2). Based on the OC-SVM model, suitable environmental conditions mirroring localities with OROV detections were found across tropical regions with scattered patches of non-suitability corresponding to areas of high altitude and around the central Amazonian region. In the Caribbean, regions with OROV transmission-risk included Puerto Rico, Dominican Republic, and Haiti. In North America, areas of OROV transmission risk were detected in southwestern Mexico and the coastal regions of Baja California and Baja California Sur (Figs. 3 and 4). OROV transmission risk in the U.S. was restricted to focalized coastal regions of California and western Florida. Areas that until today lack OROV reports but are of risk according to our model included eastern Bolivia, Paraguay, and Uruguay (Figs. 3 and 4).

### Occurrence contribution to the final model

The influence of every occurrence on OROV risk mapping was assessed through a Jackknife approach revealing two localities with OROV transmission that greatly influenced the final forecast (e.g., exclusion of those two sites decreased > 10% the area predicted; Fig. 4 and Table 3). In the northern locality, annual mean temperature and annual mean specific humidity (i.e., BIO1 and BIO12) were higher. Conversely, the highly influential locality at the south (i.e., northern Argentina) showed annual mean temperature values similar to those found across the bulk of occurrences but with a higher variation expressed as higher temperature range (i.e., BIO1 and BIO7; Fig. 4 and Table 3).

**Table 3:** Summary statistics of environmental predictors at identified localities

Environmental predictors	Localities	Mean	Standard deviation
BIO1	Northern site	26.2	0.52
	Southern site	22.13	4.03
	All sites	22.92	2.11
BIO7	Northern site	7.8	0.62
	Southern site	36.65	1.91
	All sites	19.85	0.88
BIO12	Northern site	0.019	0.00073
	Southern site	0.0090	0.00079
	All sites	0.013	0.00046

Summary statistics obtained from a 100 km buffer around localities used in this study. We compared the environmental values of those points decreasing the geographical prediction in more than 10% (red points Fig. 4) versus the average across all sites. BIO1: Annual mean temperature, BIO7: Temperature annual range, BIO12: Annual mean specific humidity

### The role of vegetation

**Fig. 5:**
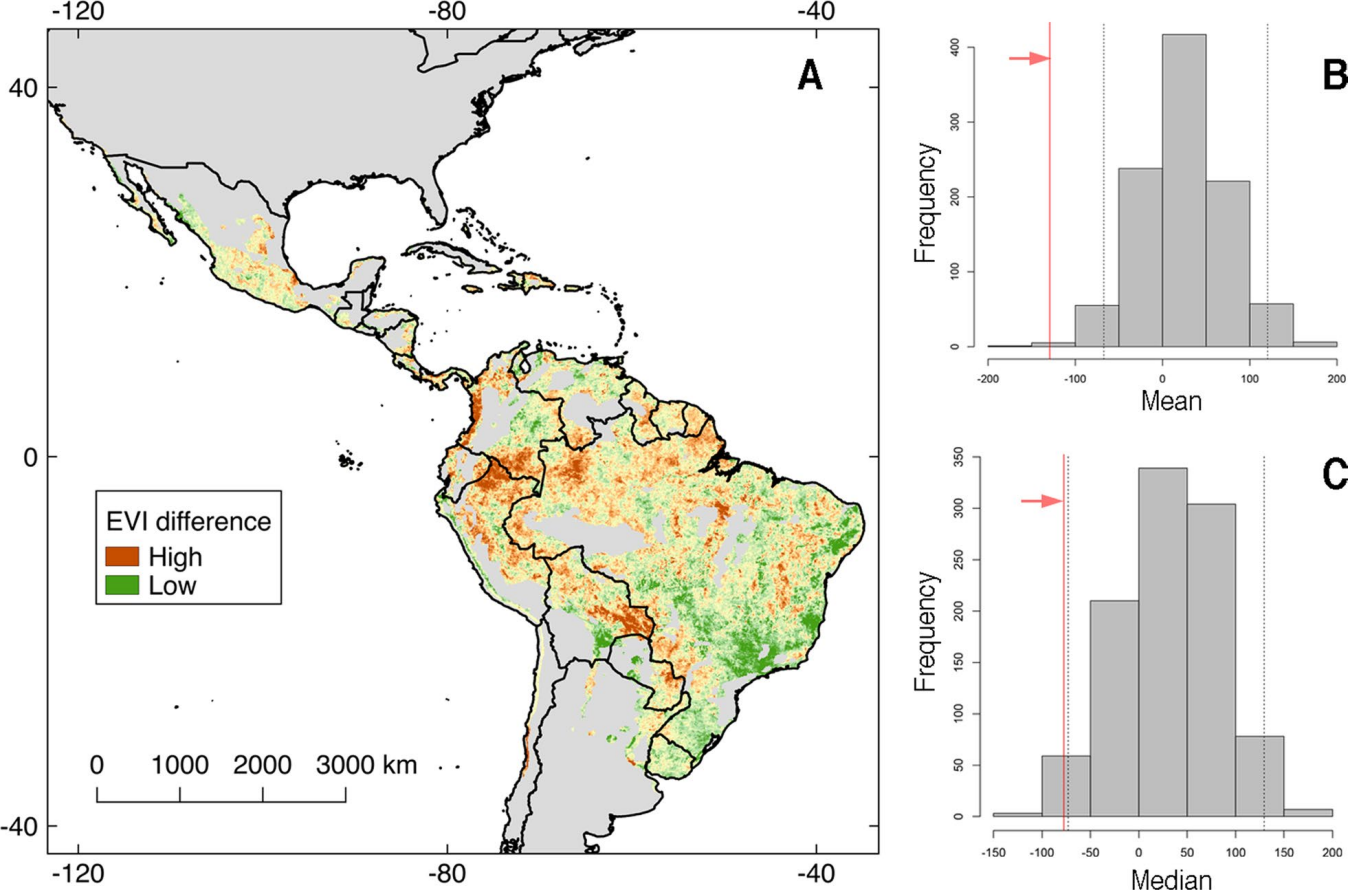
Enhanced vegetation index (EVI) values across Oropouche virus (OROV) transmission risk map. Vegetation difference between 2019 and 2003 from the MOD13A2 version six products from the MODIS sensor from the TERRA satellite. **A** Regions with low (green) and high (brown) EVI difference are depicted inside the OROV transmission risk map. **B** Results of a randomization test using the mean of EVI values from the 35 OROV occurrences (red line) in comparison with 1000 replicates of 35 random draws across the OROV transmission risk map. Note that observations (arrow) fall outside the non-significant region (dashed lines) **C** Same as** B** but using the median as observed statistic. Shapefile of the Americas obtained from NaturalEarth (https://www.naturalearthdata.com/) and maps developed with QGIS 2.18 ‘Las Palmas’ and Adobe Photoshop Elements

Randomization tests revealed that vegetation loss, measured using NDVI and EVI, increased the likelihood of OROV transmission risk (Fig. 5 and Additional file 2). When analyzing EVI values from OROV occurrences versus random points, we found a significant difference using either the mean or median as evaluating statistics (Fig. 5). NDVI values of OROV occurrences were significantly different than the null for the mean values (Additional file 2), suggesting that EVI values within the potential OROV distribution are more consistent to the presence of non-parametric data. Regions of vegetation loss include the western coast of Colombia, Amazonian regions of Ecuador, Colombia, and Peru, and eastern Bolivia and Paraguay (Fig. 5).

### Human population at risk

**Fig. 6:**
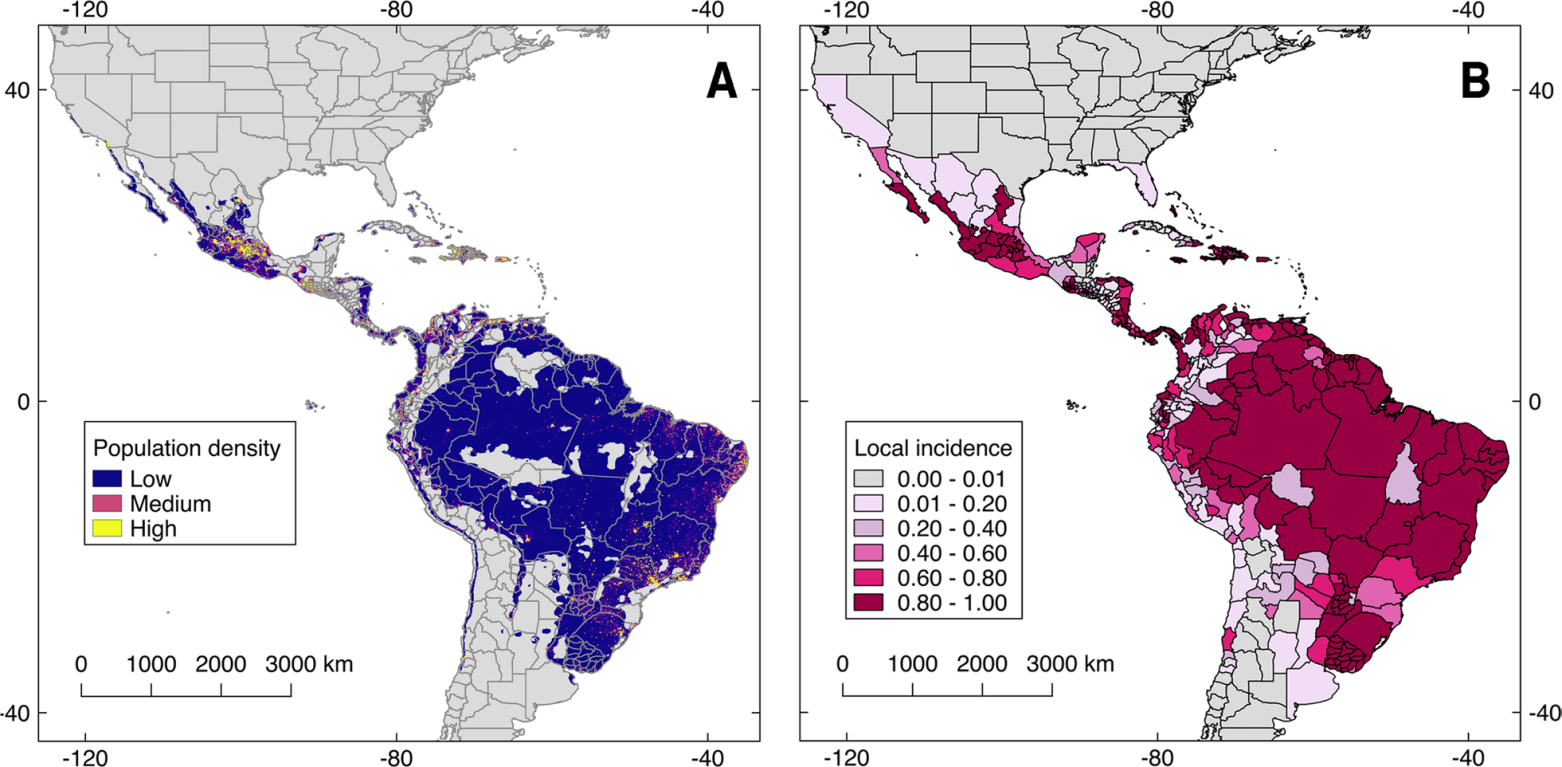
Population at risk of Oropouche virus (OROV) transmission. We estimated the population at risk of OROV transmission using the population for 2020 via the WorldPop unconstrained data for the Americas (https://www.worldpop.org/geodata/summary?id=24777; **A** and the OROV distribution obtained through one-class support vector machines (OC-SVM) hypervolumes (Fig. 3 and 4). Our analysis suggests that 4,920,600 million people overlap with OROV transmission risk map. The right map depicts local incidence, as the proportion of population pixels suitable according to our model, divided by the total population pixels available in each province/state (**B**). Data for developing this map is available at the Additional file 4. Shapefile of the Americas obtained from NaturalEarth (https://www.naturalearthdata.com/) and maps developed with QGIS 2.18 ‘Las Palmas’ and Adobe Photoshop Elements

We calculated the potential local incidence of OROV per province, department, or state across the areas predicted of risk according to the best model (Fig. 3 and 4). We found that approximately 4,920,600 people live in areas predicted suitable for OROV transmission in the Americas (Fig. 6). Regions that might be at higher risk of case detection included the coasts of Ecuador, Colombia and Venezuela, Panama, central Mexico, Brazil, and eastern Bolivia (Fig. 6). By restricting the analysis only to areas of model agreement between PCs and climatic predictors, we found that approximately 2,393,803 people living on OROV potential distributional area (Additional file 2 and 4).

## Discussion

Mapping disease transmission risk for data-limited emerging diseases might help guide surveillance systems [64–66]. To the best of our knowledge, this is the first time a risk map for Oropouche fever is proposed through the lenses of ecology and disease biogeography [67]. We evaluated the effects of predictors, presence-only algorithms, model calibration regions, and individual occurrences on the prediction of OROV transmission risk in the Americas (see “Study design” section and Fig. 1).

One-class support vector machines and convex hull hypervolumes might have a role on preliminary approaches to the distribution of poorly known pathogens. Limited data are a common trend for emerging infectious diseases of wildlife origin [11]. Without enough data, representation of environments and areas of risk might be highly uncertain because the identification of all the species participating in the disease system may be hindered. The dependency of species distribution models to the calibration region ***M*** is pervasive across every modeling step [35, 68, 69]. We found that for the presence-only algorithms explored in this study, the definition of ***M*** has little influence on the geographical projection of the final model, especially when using climatic predictors (Additional file 2). Although the size of the calibration region modified the size of the environmental volume estimated (Table 1), the geographic output was minimally affected (Fig. 3 and Additional file 2).

Model evaluation in species distribution modeling is a topic of continuous debate. Evaluation metrics are dependent on the calibration region ***M*** and therefore subjective to a particular research question, assumptions of dispersal, and data availability [36, 37, 70, 71]. To overcome the effect of ***M*** in the risk mapping process, we implemented a bootstrap approach to select a particular model based on maximizing sensitivity and, therefore, model performance [33, 52]. We found that performance metrics were similar despite the differences in the model calibration region, especially when evaluated in the environmental space (Tables 1 and 2). Models developed with OC-SMV hypervolumes and PCs outperformed models developed with climatic predictors (Tables 1 and 2). At least two reasons might explain this pattern. First, PCs recover more information than the three uncorrelated climatic predictors because PCs summarize information from 15 predictors. Second, hyperellipses built around occurrences in the environmental space through OC-SVM hypervolumes are less constrained to identify similar regions than convex hulls [42]. It is worth mentioning that although minimal in this study, geographic projections using PCs are still sensitive to model calibration region because transformed variables recover information proportional to the amount of data available [32, 72]. Thus, three PCs from smaller areas will recover more information than the same number of PCs from larger areas (Table 1).

Our analytical approach is derived from ecological niche theory aiming to recover a proxy of the realized ecological niche of the pathogen, which we quantified in an n-dimensional environmental space. Traditional correlative ecological niche approaches are generally data-hungry to allow the working algorithm to characterize response curves of the target organism to the surrounding environments [73, 74]. Because OROV is a data-poor disease system, we employed hypervolumes to represent the environmental conditions of OROV occurrences across environments in the Americas. Moreover, due to the lack of information of OROV, both in terms of case occurrences and the unknowns regarding its sylvatic cycle (i.e., vectors and reservoirs; [4]), a ‘black box’ approach was used to recover the conditions where human outbreaks occur (Figs. 1 and 2). In this regard, the OC-SVM and convex hull presence-only algorithms used in this study [23, 75, 76] could be implemented in ‘black-box’ disease risk mapping.

The final Oropouche fever risk map suggests that ~ 5 million people overlap with areas of OROV transmission risk (Fig. 6). The predicted hotspots of OROV transmission risk denote the potential distribution of the disease from the southern U.S. to Uruguay (Figs. 3 and 4). These results may be overestimating OROV impacts considering that OROV outbreaks have not been reported outside South America since the 1990’s, when OROV was reported in Panama [4]. Nevertheless, the presence of vectors across the continent, including in North America (i.e., *Cu. paraensis* and *Cx. Quinquefasciatus*), reveals the latent threat for future OROV emergence across the areas predicted [3, 7, 31, 51]. Alternatively, OROV may already be present in multiple regions shown here, yet it has not been detected due to the lack of epidemiological awareness and precise clinical or laboratory diagnosis [5], as exemplified with the false negative OROV cases reported in Ecuador in 2018 [51].

The risk map of Oropouche fever transmission represented using a chropleth map as a proxy of local incidence (Fig. 6 and Additional file 2) is an effort to translate our findings (Figs. 3 and 4) to a ready-to-examine output accounting for administrative units, specifically because epidemiology and public health interventions are usually implemented over well-delimited political units [77]. For example, the present map highlights how only two Brazilian states might be considered with less risk of disease detection in comparison to the rest of the country (Fig. 6). Historical Oropouche fever outbreaks in Brazil since the 1960’s show how the disease has been detected across the entire country [4]. Unsurprisingly, Oropouche fever is considered the most common VBD after dengue in Brazil [6, 22]. Thus, febrile syndromes of unknown etiology across the regions identified by our models should prompt clinicians to consider OROV in the differential diagnosis of suspected arboviral febrile illnesses.

The regions identified as environmental outliers for OROV outbreaks were registered at Mazagão, Amapá, Brazil as the northern site [78], and Palmasola, Jujuy, Argentina in the south [79], both driven by differences of temperature and humidity against the average of the bulk of OROV occurrences (Fig. 4). These variables have been found to be crucial in determining vector population dynamics and parameters of disease transmission in other VBDs [28, 30]. Further investigation of outbreaks in these areas might inform on climatic or other specific ecological particularities contributing to the emergence of OROV outside well-known endemic regions.

Randomization tests showed a decrease on vegetation in OROV occurrences in comparison with surrounding areas (Fig. 5), which could be interpreted as more habitat loss in sites with OROV emergence. This difference was consistent for EVI across the two statistics used for the development of the randomization test. For NDVI, only the mean showed a significant difference between OROV outbreaks versus random localities (Additional file 2). We suggest that within OROV potential distribution, EVI values were more stable to the presence of non-parametric data and should be used to detect subtle changes on areas with dense vegetation as in the Amazon region [2, 80–82]. Likely mechanisms linking vegetation loss and OROV outbreaks include the increased contact between humans and infected arthropods in deforested areas, and impacts in the assemblage of wildlife species affecting the distribution and abundance of vectors [2, 83]. Areas at risk of OROV emergence and with increased ecosystem degradation might be good targets for active surveillance for early OROV detection. For example, an endemic case was recently reported in Turbaco, Colombia [84], an area with increased ecosystem degradation in an area of OROV transmission risk predicted here (Fig. 5).

As global connectivity increases, the risk of OROV translocation beyond the Americas is a probability that should be highlighted, especially due to the global distribution of *Cu. quinquefasciatus* [85]. Although vector capacity of this mosquito is still being discussed, reports have shown its capacity to host and transmit the virus [8, 9]. More importantly, uncertainties around OROV reservoir should also be acknowledged. Apart from *Bradypus tridactylus* and *Callithrix penicillata*, biomarkers (i.e., molecular or antibodies) of OROV have been detected in mammals such as *Allouatta caraya*, *Sapajus alloata*, and *Proechimys* sp., and birds from the families Formicariidae, Fringillidae, Thaurapidae, and Columbidae [4]. Experimental transmission studies are needed to assess the capacity of these vertebrates to serve as reservoirs or amplification hosts. Nevertheless, a priori, OROV seems to be a pathogen with broad capacity of infection, which is another argument to improve surveillance and research to anticipate its establishment in the Americas or other continents.

Despite the comprehensive methodological approach, limitations of the present study include a limited number of occurrences for model development, poor understanding of the sylvatic cycle of the disease (e.g., wildlife reservoirs), and the lack of independent testing data [16, 50]. These three components are inherent to any species distribution model applied to emerging tropical infectious diseases [12, 16]. We, however, tried to advert the scarcity of occurrences with an exhaustive literature review [4] and using variable selection methods that better fit with the available case records (Fig. 1). Model evaluations in the field are seldom developed due to the inherent lacking of resources for epidemiological surveillance in the absence of outbreaks. A next frontier in OROV research should consider assess the virus circulation in diverse species, areas, and landscape conditions, especially before outbreaks occur as a means for more proactive—instead of reactive—OROV investigations guided by our mapping efforts.

## Conclusions

Hypervolume modeling can be a first step towards unveiling ecological and geographic patterns of disease transmission risk. Our studies revealed that between 2 to 5 million people might be at risk of exposure to OROV across the Americas and future outbreaks might be related to vegetation loss in the region. Our preliminary OROV risk map offers opportunities to identify areas and ecosystems for future research including investigations into the likely OROV wildlife reservoirs and designing disease prevention and monitoring plans. Oropouche fever is an emerging infectious disease of wildlife origin with considerable epidemic potential.

## Supplementary Information

**Additional file 1:** Occurrences used for developing species distribution models.

**Additional file 2: Fig. S1.** Pearson correlation matrix across 15 bioclimatic predictors.** Fig. S2.** Study areas used for model calibration and evaluation.** Fig. S3.** Comparison of geographical predictions across South America.** Fig. S4.** Uncertainty of models developed with hypervolumes.** Fig. S5.** Potential distribution of Oropouche fever based on convex hulls.** Fig. S6.** Uncertainty of models developed with convex hulls.** Fig. S7.** Normalized difference vegetation indexvalues across the potential distribution of Oropouche virus.** Fig. S8.** Population at risk of Oropouche virusinfection on areas of model agreement.

Additional file 3. List of R packages used for the development of the Oropouche fever models.

Additional file 4. List of provinces/states at risk of Oropouche fever according to the final model and regions of model agreement.

## Data Availability

All data and codes to replicate the study can be found within the present manuscript and repositories cited.

## Abbreviations

EVI: Enhanced vegetation index
*M*: Sensu for study area in species/ecological niche models
MODIS: Moderate Resolution Imaging Spectroradiometer
NDVI: Normalized difference vegetation index
OC-SVM: One-class support vector machines
OROV: Oropouche virus
PCA: Principal component analysis
PCs: Principal components
VBDs: Vector-borne diseases
