# Supplementary material for: Transmission risk of Oropouche fever across the Americas
Source: Infect Dis Poverty. 2023 May 6;12:47. doi: 10.1186/s40249-023-01091-2 (PMC10163756; doi:10.1186/s40249-023-01091-2)

**Transmission Risk of Oropouche Fever across the Americas**

Daniel Romero-Alvarez, Luis E. Escobar, Albert J. Auguste, Sara Y. Del Valle, Carrie A. Manore

**Additional file 2**

Information available as additional file 2:

1. Additional figures.
   1. Fig. S1: Pearson correlation matrix across 15 bioclimatic predictors.
   2. Fig. S2: Regions used for model calibration and evaluation.
   3. Fig. S3: Comparison of geographical predictions across South America.
   4. Fig. S4: Uncertainty of models developed with hypervolumes.
   5. Fig. S5: Potential distribution of Oropouche fever based on convex hulls.
   6. Fig. S6: Uncertainty of models developed with convex hulls.
   7. Fig. S7: Normalized difference vegetation index (NDVI) values across the potential distribution of Oropouche virus (OROV).
   8. Fig. S8. Population at risk of Oropouche virus (OROV) infection on areas of model agreement.

**Fig. S1. Pearson correlation matrix across 15 bioclimatic predictors.** We used a correlation matrix to select three uncorrelated environmental predictors using a threshold of 0.90; specifically we used annual mean temperature (BIO1), temperature annual range (BIO7), and annual mean specific humidity (BIO12) in subsequent analysis.


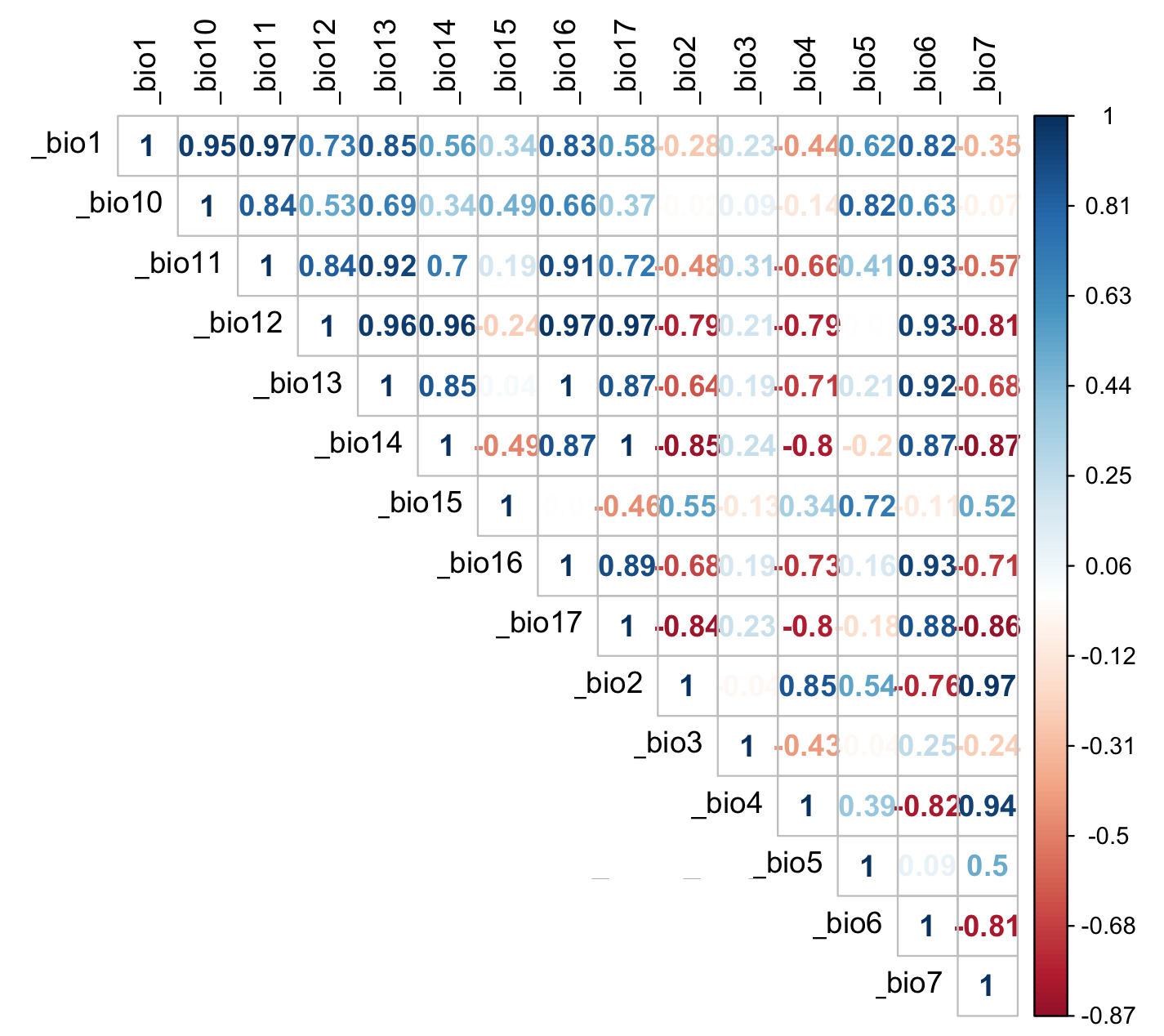


**Fig. S2. Regions used for model calibration and evaluation.** Calibration regions included a buffer using the mean of the distances between all occurrences to the geographical centroid (red), all South America (blue), and the whole American continent (black). Shapefile of the Americas obtained from NaturalEarth (<https://www.naturalearthdata.com/>) and maps developed with QGIS 2.18 ‘Las Palmas’ and Adobe Photoshop Elements.


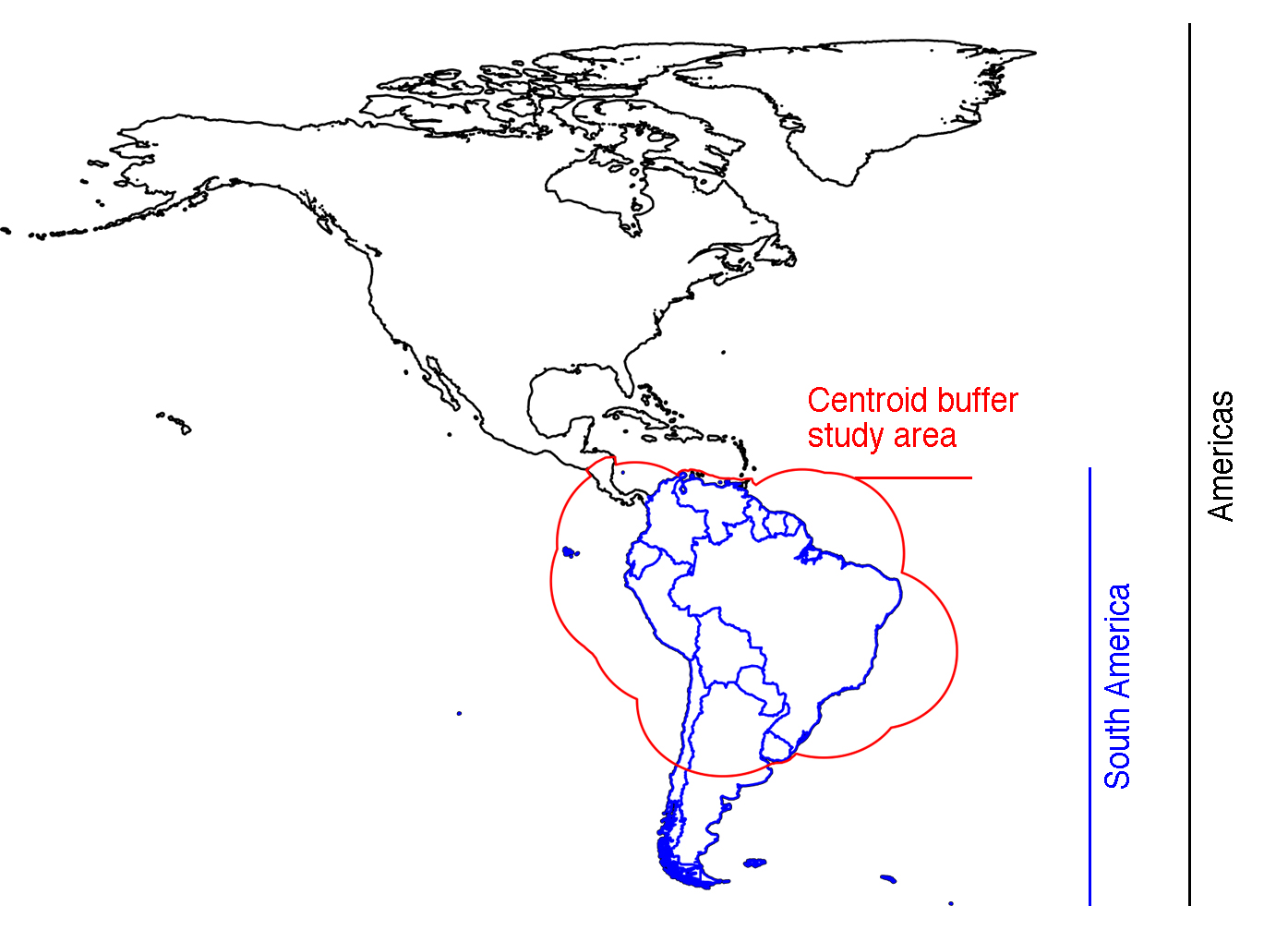


**Fig. S3. Comparison of geographical predictions across South America.** Different regions were used for model calibration (Fig. S2). While one-class support vector machines (OC-SVM) were most consistent between environmental predictors, i.e., principal components (PCs) versus climates (left panels), convex hulls predicted sparser areas of suitability with PCs and therefore showed larger variability within environmental predictors (right panels). Models based on PCs showed higher variability between algorithms (upper panels) than models built with untransformed climatic predictors, in which the difference across study areas was minimum irrespective of the selected algorithm (bottom panels). Shapefile of the Americas obtained from NaturalEarth (<https://www.naturalearthdata.com/>) and maps developed with QGIS 2.18 ‘Las Palmas’ and Adobe Photoshop Elements.


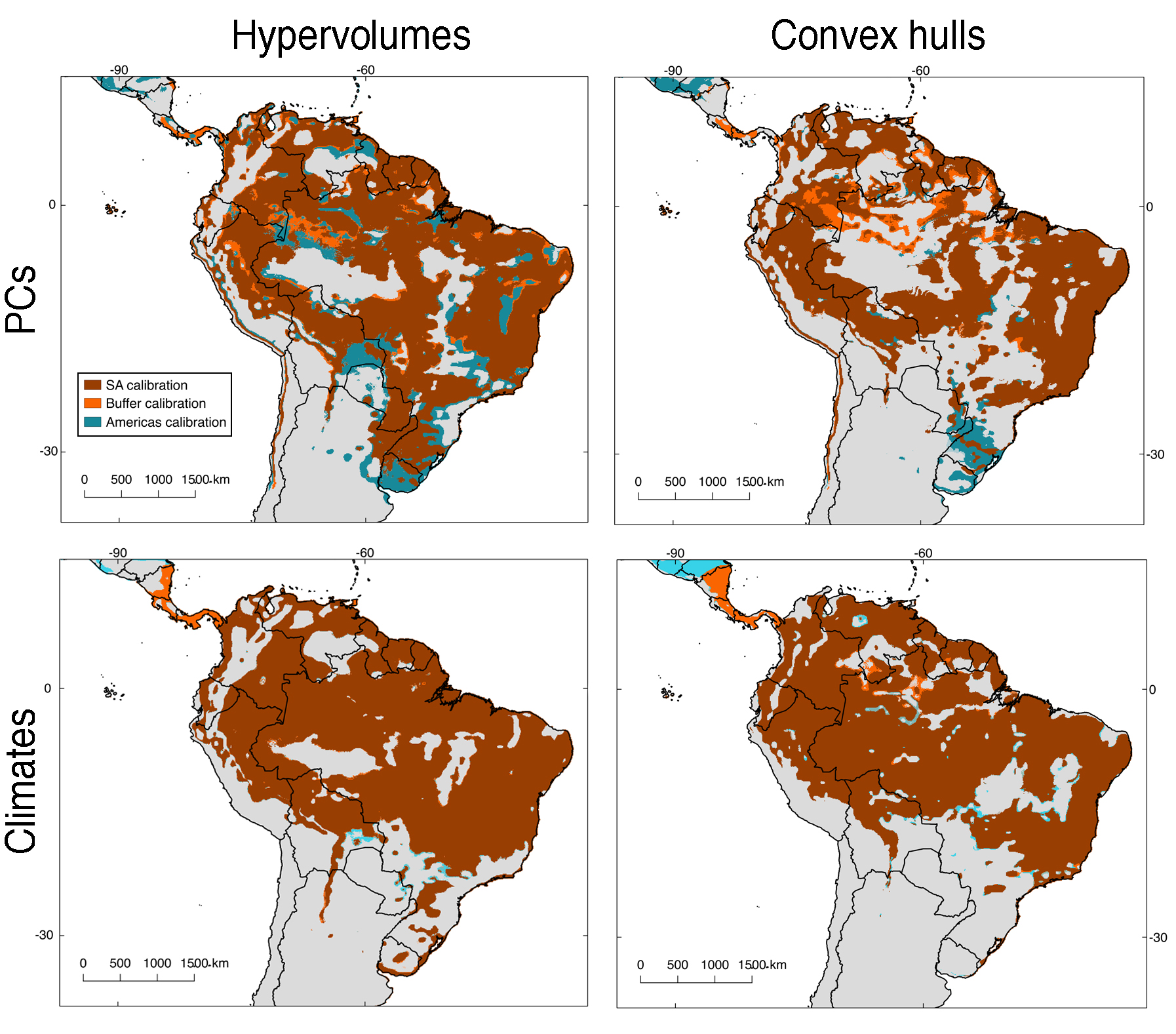


**Fig. S4. Uncertainty of models developed with one-class support vector machine (OC-SVM) hypervolumes.** Uncertainty is shown for OC-SVM hypervolumes created with principal components (PCs, **A**) and climatic predictors (**B**). For both cases, uncertainty is represented based on statistical grounds. Since the median of a bootstrap of 50 replicates was used to identify the best performing model, the 2.5% and 97.5% percentiles of those replicates represent a range were different areas were represented few times (2.5%, high uncertainty) or multiple times (97.5%, low uncertainty) in the bootstrap. Shapefile of the Americas obtained from NaturalEarth (<https://www.naturalearthdata.com/>) and maps developed with QGIS 2.18 ‘Las Palmas’ and Adobe Photoshop Elements.

**
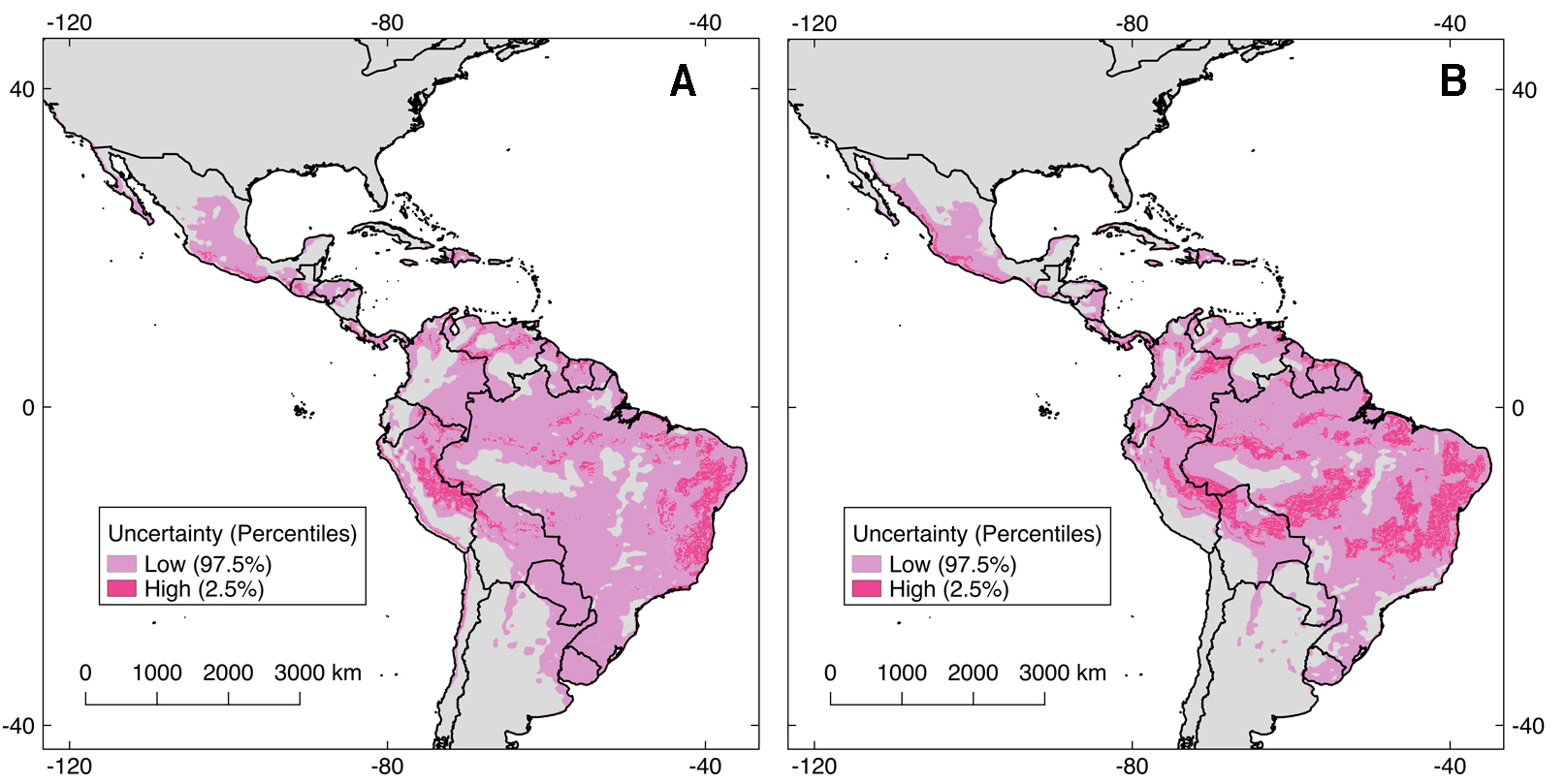
**

**Fig. S5. Potential distribution of Oropouche virus (OROV) based on convex hull hypervolumes.** Models based on convex hulls and calibrated in the Americas showed a larger disagreement between suitability suggested with principal components (PCs, **A**) and climatic predictors (**B**) The map in **C** shows areas of overlap between the suitability of both environmental predictors. Shapefile of the Americas obtained from NaturalEarth (<https://www.naturalearthdata.com/>) and maps developed with QGIS 2.18 ‘Las Palmas’ and Adobe Photoshop Elements.

**
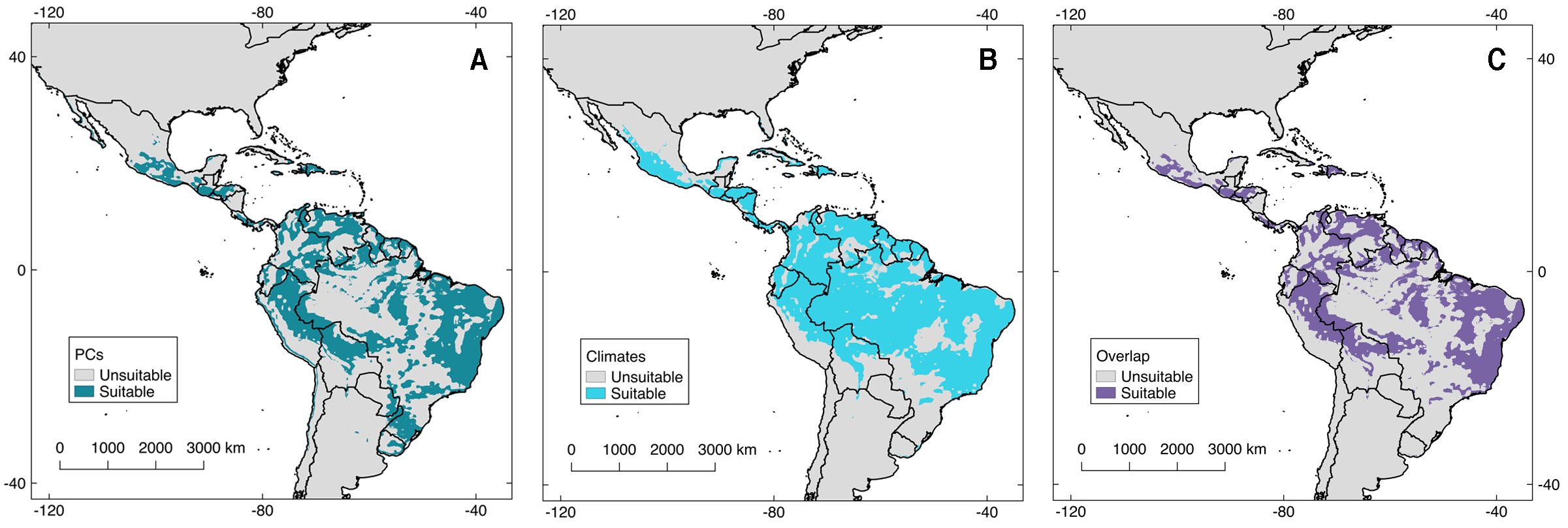
**

**Fig. S6. Uncertainty of models developed with convex hulls.** Uncertainty is shown for convex hulls created with principal components (PCs; **A**) and climatic predictors (**B**). For both cases, uncertainty is represented based on statistical grounds. Since the median of a bootstrap of 50 replicates was used to identify the best performing model, the 2.5% and 97.5% percentiles of those replicates represent a range were different areas were represented few times (2.5%, high uncertainty) or multiple times (97.5%, low uncertainty) in the bootstrap. Shapefile of the Americas obtained from NaturalEarth (<https://www.naturalearthdata.com/>) and maps developed with QGIS 2.18 ‘Las Palmas’ and Adobe Photoshop Elements.

**
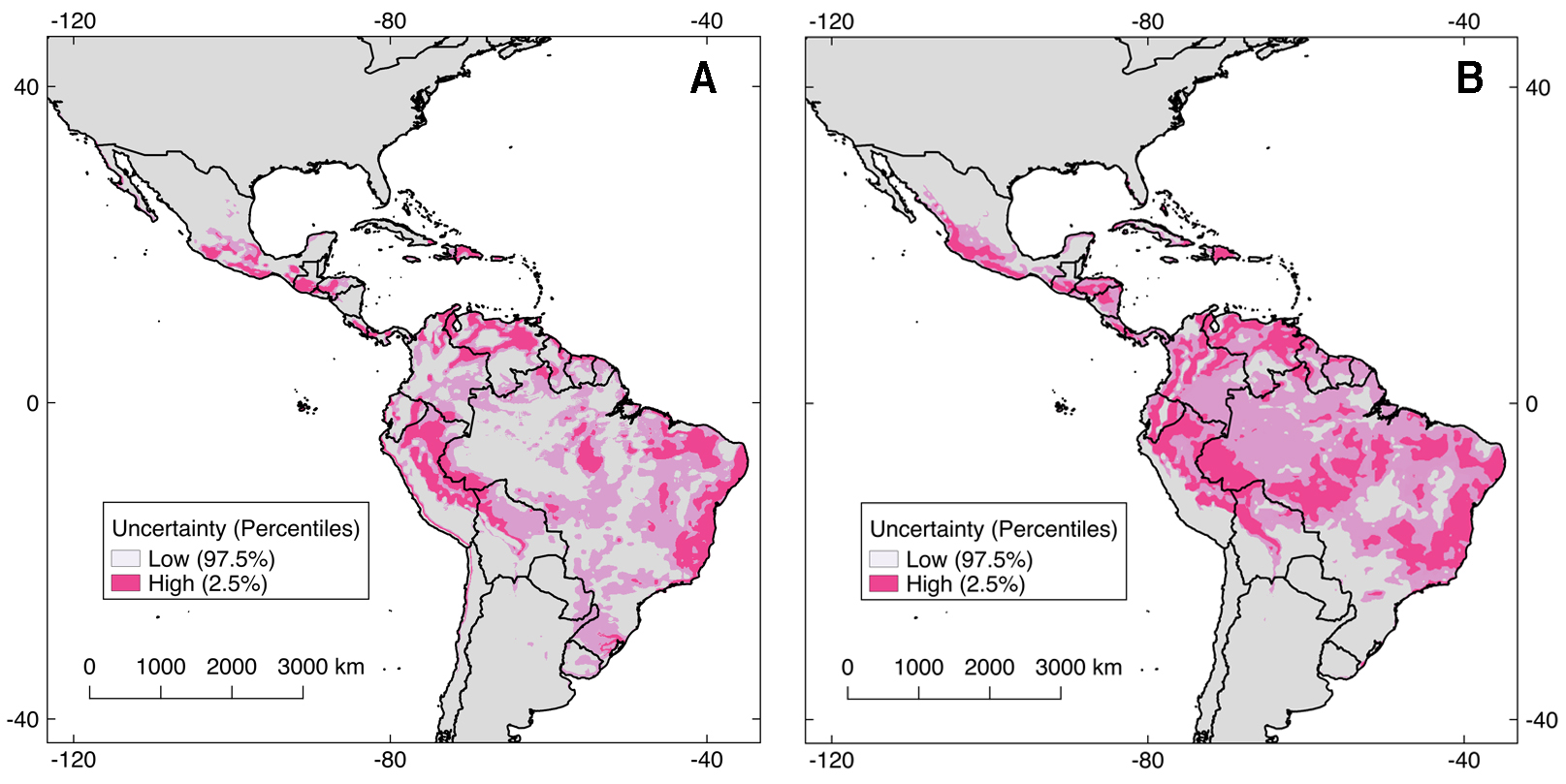
**

**Fig. S7. Normalized difference vegetation index (NDVI) values across the potential distribution of Oropouche virus (OROV) transmission risk map.** We obtained the difference between 2019 and 2003 averaged satellite images from the MOD13A2 version six products from the MODIS sensor from the TERRA satellite. (**A**) Regions with low (green) and high (brown) NDVI difference are depicted inside the potential distribution of OROV according to our models. (**B**) Results of a randomization test using the mean of NDVI values from the 35 OROV occurrences (red line) in comparison with 1000 replicates of 35 random draws across the OROV potential distribution. (**C**) Same as B but using the median as observed statistic. Notice that when using NDVI, the mean but not the median of OROV NDVI values is different from the null distribution (grey). Shapefile of the Americas obtained from NaturalEarth (<https://www.naturalearthdata.com/>) and maps developed with QGIS 2.18 ‘Las Palmas’ and Adobe Photoshop Elements.


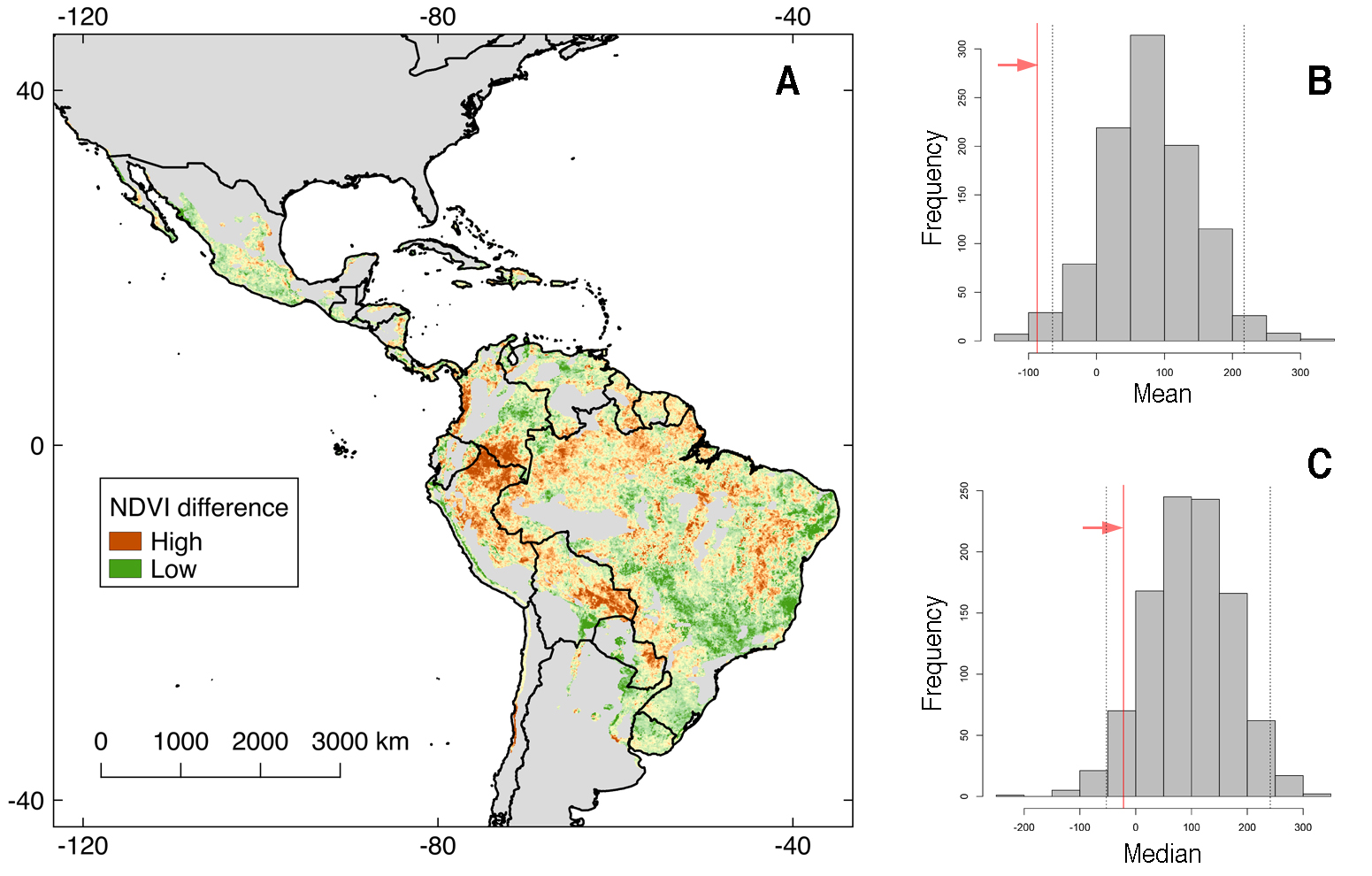


**Fig. S8. Population at risk of Oropouche virus (OROV) transmission on areas of model agreement.** By using only the overlapping distribution between one-class support vector machine (OC-SVM) hypervolume models built with principal components (PCs) and climatic predictors (Fig. 3 and 4, main text) we estimated the population at risk of OROV using the population for 2020 via the WorldPop unconstrained data for the Americas (<https://www.worldpop.org/geodata/summary?id=24777>; **A**). Our analysis suggests that 2,393,803 million people overlap with OROV potential distribution (**B**). The map depicts local incidence, calculated as the proportion of population pixels suitable according to our model, divided by the total population pixels available in each province/state (**B**). Data for developing this map is available at the Additional file 4. Shapefile of the Americas obtained from NaturalEarth (<https://www.naturalearthdata.com/>) and maps developed with QGIS 2.18 ‘Las Palmas’ and Adobe Photoshop Elements.


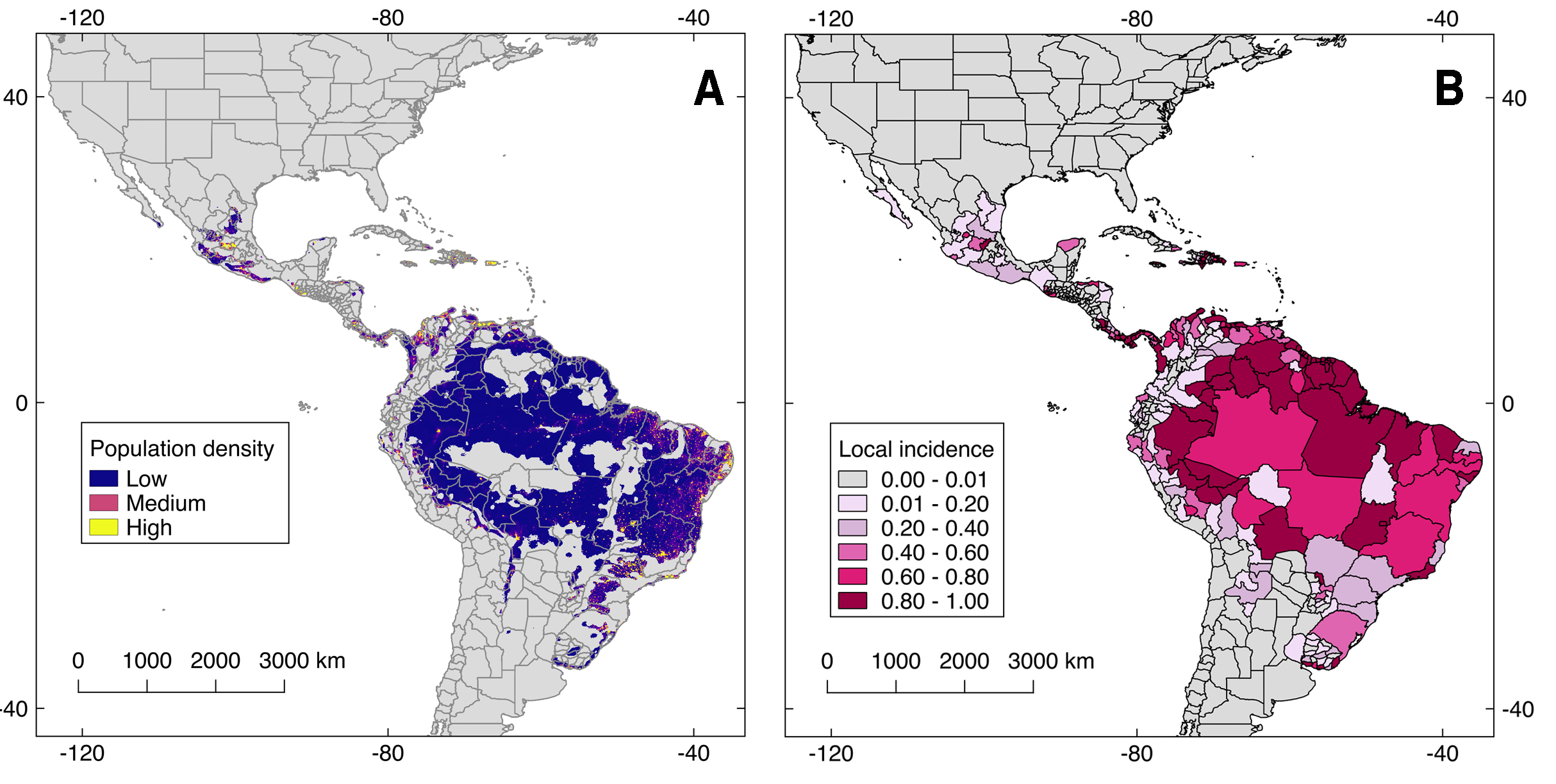

Supplement: Supplementary file 2 — Additional file 2: Fig. S1. Pearson correlation matrix across 15 bioclimatic predictors. Fig. S2. Study areas used for model calibration and evaluation. Fig. S3. Comparison of geographical predictions across South America. Fig. S4. Uncertainty of models developed with hypervolumes. Fig. S5. Potential distribution of Oropouche fever based on convex hulls. Fig. S6. Uncertainty of models developed with convex hulls. Fig. S7. Normalized difference vegetation indexvalues across the potential distribution of Oropouche virus. Fig. S8. Population at risk of Oropouche virusinfection on areas of model agreement. [file 40249_2023_1091_MOESM2_ESM.docx]
